# The Effects of Swimming Training in Cold Water on Antioxidant Enzyme Activity and Lipid Peroxidation in Erythrocytes of Male and Female Aged Rats

**DOI:** 10.3390/ijerph16040647

**Published:** 2019-02-22

**Authors:** Anna Lubkowska, Iwona Bryczkowska, Izabela Gutowska, Iwona Rotter, Natalia Marczuk, Irena Baranowska-Bosiacka, Giuseppe Banfi

**Affiliations:** 1 Department of Functional Diagnostics and Physical Medicine, Faculty of Health Sciences, Pomeranian Medical University in Szczecin; Żołnierska 54, 71-210 Szczecin, Poland; annalubkowska@gmail.com (A.L.); ibryczkowska@gmail.com (I.B.); 2 Department of Biochemistry and Human Nutrition, Faculty of Health Sciences, Pomeranian Medical University in Szczecin; Broniewskiego 24, 71-460 Szczecin, Poland; 3 Department of Medical Rehabilitation, Faculty of Health Sciences, Pomeranian Medical University in Szczecin, Żołnierska 54, 71-210 Szczecin, Poland; iwrot@wp.pl; 4 Department of Microbiology, Immunology and Laboratory Medicine, Faculty of Medicine with English Language Teaching Department, Pomeranian Medical University in Szczecin; Powstańców Wielkopolskich 72, 70-111 Szczecin, Poland; n.marczuk03@yahoo.pl; 5 Department of Biochemistry, Faculty of Medicine with English Language Teaching Department, Pomeranian Medical University in Szczecin; Powstańców Wielkopolskich 72, 70-111 Szczecin, Poland; irena.bosiacka@pum.edu.pl; 6 Laboratory of Experimental Biochemistry and Molecular Biology, IRCCS Istituto Ortopedico Galeazzi, Via Riccardo Galeazzi, 4, 20161 Milano, Italy; banfi.giuseppe@fondazionesanraffaele.it; 7 Vita-Salute San Raffaele University, Via Olgettina Milano, 58, 20132 Milano, Italy

**Keywords:** antioxidant enzymes, cold swim, elderly rats, lipids peroxidation

## Abstract

The aim of this study was to verify whether eight-week-long swimming exercise training would evaluate the level of selected indicators of the pro-oxidant/antioxidant status in response to cold water in comparison with swimming under thermoneutral conditions in sedentary male and female elderly rats. The exercise-trained groups swam four min/day and five days a week during eight weeks of housing. Exercise was performed by swimming in glass tanks containing tap water maintained according to group at 5 °C and 36 °C. At the end of treatment (48 h after the last session), all rats were anaesthetized. The level of chosen biomarkers of oxidative stress and antioxidant enzyme activity was determined in the red blood cells and plasma. The results of study show that female rats seem to be better adapted to changing thermal conditions of the environment, developing not only morphological, but also antioxidant, defense mechanisms, mainly in the form of increased erythrocyte superoxide dismutase (SOD) activity and glutathione (GSH) concentration to restore the pro-oxidant/oxidant balance of the organism. Significantly higher concentrations of GSH were observed in the female rats of the group swimming in cold water (by 15.4% compared to the control group and by 20.5% in relation to the group of female rats swimming at 36 °C). In the group exposed to swimming training exercise in cold water, a significantly higher activity of SOD1 (by 13.4%) was found compared to the control group. On the other hand, the organs of ageing male rats show a reduced capacity to increase the metabolic response to low temperatures compared to female ones. In addition, it was demonstrated that cold exposure leads to an increase in lipid peroxidation in tissues. On the other hand, the repeated exposure to low levels of oxidative stress may result in some adaptive changes in organisms that help them to resist stress-induced damage.

## 1. Introduction

The ageing process is one of the main unmodifiable risk factors for life-threatening conditions, reducing immunity and common diseases, including type 2 diabetes, cardiovascular and respiratory system disease, neurodegenerative disease, and cancer [1,2]. As ageing progresses, lipid and protein oxidation and unrepaired damage to DNA accumulated over a lifetime may cause cellular senescence [3], which is postulated as a theory of ageing. Moreover, both the free radical theory and oxidative stress are of the hypotheses associated with ageing, suggesting that oxygen-free radicals are formed endogenously from normal oxygen-utilizing metabolic processes and play an essential role in this process. The irradiation of living things, known to induce the formation of free radicals, shorten their lifespan and produce changes that resemble ageing [4,5]. Involvement of free radicals in ageing has increased progressively and is becoming one of the more plausible theories of the ageing process [6,7]. It is believed that the antioxidant defense is generally weakened in old age. One of the well-known physiological conditions characterized by the increased production of free radicals is exercise [8,9]. Many reports confirm that exercises cause oxidative stress through free radical generation [10,11] and a decrease in the levels of antioxidant enzymes in different tissues and organs [12,13,14,15], but different intensities and forms of exercise result in different levels of biological stress. The production of free radicals increases with the increase in oxygen consumption and oxidative phosphorylation. This is directly corelated with intensity of exercise. Increases in blood levels of lipid hydroperoxides, F2-isoprostanes (Iso-P), malondialdehyde (MDA) and conjugated dienes were reported after exercise [16,17]. On the other hand, the antioxidant enzymes activity and its concentration in organs, which constitute a defense mechanism against free radicals, are also affected by the exercise. It is also commonly accepted that regular physical effort leads to an increase in the activities of antioxidant enzymes, especially in muscles [18,19,20,21]. Another strong physiological and psychological environmental stressor causing the physical and chemical thermoregulatory response is exposure to a cold environment and cold stimulation in different form of therapy [22]. Nasu et al. proposed that repeated cold stress might serve as a useful model for study of the mechanism of chronic muscle pain and its treatment [23]. 

Water immersion is used in some cultures as a means of health restoration by athletes after training sessions for reducing muscle pain or accelerating recovery of fatigued muscle, as well as by older people in their lifestyle modification. Physiological changes are attributed principally to the effects of hydrostatic pressure and temperature of exposure. Exercise in a cold environment has provided significant evidence pointing toward greater use of fat and increased VO_2_ demand [24,25], and leads to immediate and long-term physiological and biochemical responses, including both hormonal and metabolic reactions, being documented in experiments on animals and humans [26,27,28]. Cold water immersion decreases skin, subcutaneous, muscle, and sometimes even rectal temperature [29] in response to increased oxygen consumption and metabolism to maintain core temperatures. The effect of acute and/or chronic exercise on oxidative stress and antioxidant systems in old organisms was investigated in a number of studies [11,30,31,32]. However, only a few studies confirmed that cold water swimming results in oxidative stress in humans, but repeated immersions in cold water may enhance immune responses and improve antioxidant protection [17,33,34,35].

Research is lacking on the potential impact of repeated low-intensity exercise in a cold-water environment on the pro-antioxidant balance in the process of ageing. It could be assumed that repeated cold-water exercise induces a physiological change of an adaptive nature as an improved antioxidant parameters in the ageing process. In order to verify this hypothesis, we carried out research in which we evaluated the level of selected indicators of the pro-oxidant/antioxidant status in response to cold water in comparison with swimming under thermoneutral conditions in sedentary male and female elderly rats. Although there are many parameters to quantify oxidative stress, it appears that no single biomarker best assesses oxidative stress and that the measurement of multiple biomarkers is required to confirm the pro-oxidant/antioxidant status. Following this knowledge, the aim of our work was to examine the effects of swimming training in cold water on antioxidant enzyme activity and lipid peroxidation in erythrocytes of male and female aged rats. We decided to determine the changes in both erythrocyte and plasma concentrations of one of the best available biomarkers of lipid peroxidation in vivo, i.e. 8-isoprostane [36] and malonyldialdehyde (MDA) as judged by the generation of thiobarbituric acid reactive substances (TBARS). With regard to the antioxidant status, our analysis concerned the changes in reduced (GSH) and oxidised (GSSG) glutathione, taking into account the GSH/GSSG ratio and components of the enzymatic antioxidant system: Superoxide dismutase (SOD1 or CuZn-SOD; E.C.1.15.1.1), catalase (CAT; E.C.1.11.1.6), glutathione peroxidase (GPx; E.C. 1.11.1.9), glutathione reductase (GSSG-R; E.C.1.8.1.7) and glutathione S-transferase (GST; E.C.2.5.1.18) in erythrocytes. 

## 2. Material and Methods

### 2.1. Animals

**Figure 1:**
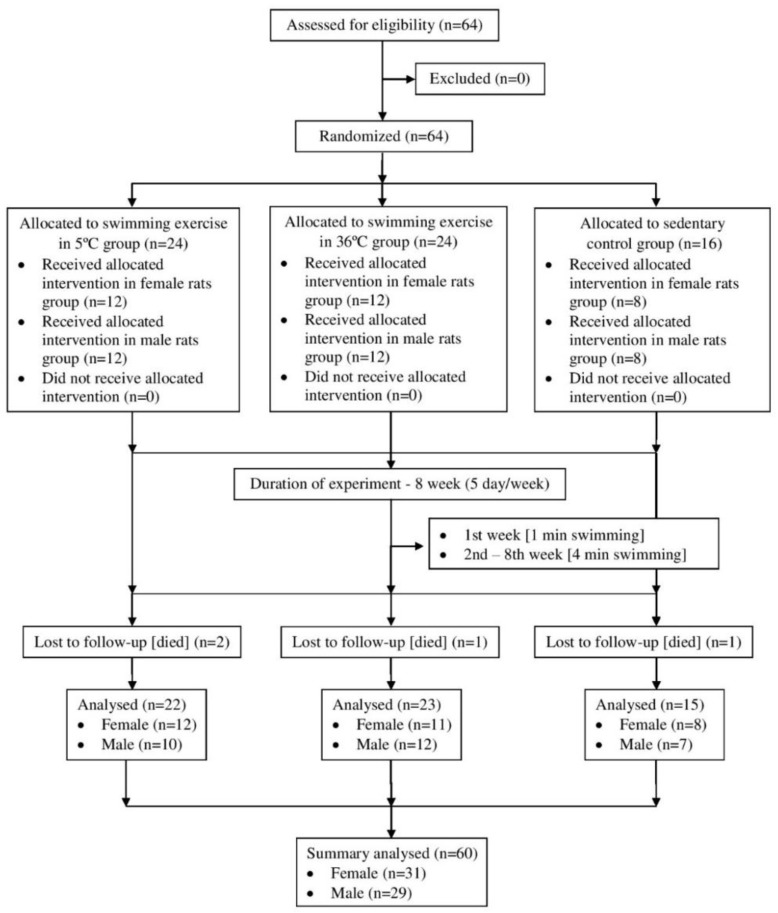
Protocol of experiment.

Sixty-four 15-month-old male and female albino Wistar rats were randomly divided into three groups. The first group (old sedentary control) was composed of male (*n* = eight) and female (*n* = eight) rats. The second group included old rats swimming in cold water (5 ± 2 °C; *n* = 12 male rats; *n* = 12 female rats). The third group were rats swimming in thermo-neutral water (36 ± 2 °C; *n* = 12 male rats; *n* = 12 female rats). All rats were given standard rat chow and tap water ad libitum and were housed two to four per cage at 23 ± 2 °C, with air relative humidity of 40% and a 12:12 h light/dark cycle. One animal from the old control and three animals from the old-swimming group died before the end of the experiment. The study was approved by the Local Ethical Committee on Animal Experimentation (Decision No. 38/2015). The protocol of experiment is presented in Figure 1.

### 2.2. Exercise Procedures

Prior to the commencement of the experiment, preliminary trials were carried out in order to determine the right duration of time for swimming at 4 °C and to observe animal behavior during spontaneous physical activity in cold and thermoneutral water. After careful analysis from the preliminary study, it was determined that, in order to eliminate the possibility of discontinuing active swimming, the animals must be placed in the tank no more than two individuals at a time, otherwise the rats climbed over each other and the load of physical effort was incomparable between the animals. Moreover, at the target water temperature (at 5 ± 2 °C), the animals began to drift passively after 4–5 minutes, lowering their physical activity. In order to eliminate the tendency of animals to escape, the tank was covered with a plexiglass cover with holes for air supply. Finally, in order to maintain the same activity of the animals of all groups, a maximum of only two rats were allowed to swim together and they were constantly stimulated to maintain active swimming. 

The groups, both the experimental group 5 ± 2 °C and the experimental group 36 ± 2 °C, swam four minutes a day, five days a week, during eight weeks of housing (between 9 a.m. and 11 a.m. on each training day). The duration of first swimming experience was limited to two min and increased by 0.5 min daily in the first week until it reached four min. The control old-sedentary rats were housed under the same conditions as the swimming rats and were handled as often as the exercise group. Exercise was performed by swimming in glass tanks (length 100 cm, width 50 cm, depth 50 cm) containing tap water maintained according to group at 5 ± 2 °C (cold group) and 36 ± 2 °C (thermo-neutral group). Before and after each week of the experiment, the rats were weighed. 

### 2.3. Section Procedures and Blood Preparation

At the end of treatment. all rats were anaesthetized with ketamine (10 mg/1000 g body mass) 48 h after the last session. Whole blood was quickly collected from the heart into EDTA-containing tubes in order to obtain erythrocytes and plasma. ‪

*Blood preparation:* The erythrocytes were separated by centrifugation (2600× *g*, 10 min, 4 °C), washed three times with 0.9% NaCl, chilled to 4 °C and, finally, frozen at −80 °C. Plasma was divided into aliquots and immediately deep-frozen at −80 °C until the time of analysis. Before the analysis, erythrocytes were thawed and the hemolysate of washed red blood cells was diluted with distilled water and chilled to 4°C. The activity of erythrocyte antioxidant enzymes (SOD1, CAT, GPX, GST, R-GSSG) was assessed using kinetic methods. The recorded values were calculated per hemoglobin concentration in the samples. 

### 2.4. Biochemical Analysis

In all mentioned cases, hemoglobin levels were assayed using Drabkin’s method [37].

#### 2.4.1. Assay of Superoxide Dismutase (SOD1) Activity in Erythrocyte

All used reagents were purchased from Sigma-Aldrich Sp. z o.o. (Poznań): (-) epinephrine, HCl, 0.05 M Na_2_CO_3_/NaHCO_3_ buffer (pH 10.2), EDTA-Na_2_, chloroform, ethanol –and were used to SOD extraction. In a test tube, a mixture of hemolysate, chloroform and ethanol solution (3:5; v/v) and distillated water were combined. Afterwards, the mixture was energetically vortexed and centrifuged (5 min; 4 °C; 3824 g). Next to the SOD extract, the Na_2_CO_3_/NaHCO_3_ buffer and adrenaline were added and the mixture was incubated for three min at 37 °C. The analysis of SOD1 activity was performed using a UV/VIS Lambda 40 spectrophotometer (Perkin-Elmer). The analysis of the study materials’ absorbency was performed over five min at a wavelength of 320 nm (at 30 °C). SOD1 activity was calculated per 1 g of RBC hemoglobin. Sensitivity of the assay was 0.1 U/mL, specificity 97%, while coefficient of variation was lower than 4% [38].

#### 2.4.2. Assay of Catalase (CAT) Activity in Erythrocyte

Reagents (50 mM phosphoric buffer, pH 7.0 (KH_2_PO_4_, Na_2_HPO_4_x12 H_2_O), H_2_O_2_ 30 mM) were purchased from Sigma-Aldrich Sp. z o.o. (Poznań). Hemolysate was diluted 500-fold using a 50 mM phosphoric buffer. Catalase activity was analysed using a UV/VIS Lambda 40 spectrophotometer (Perkin-Elmer). Absorbance measurements of the study sample (hemolysate and 30 mM H_2_O_2_ solution) were performed within 30 sec at a wavelength of l240 nm (at 30 °C). RBC catalase activity was determined based on the calibration curve, obtained as a result of assays performed for several solutions of the catalase activity pattern (Oxis Research, USA). Catalase activity was calculated per 1 g of the erythrocytes’ hemoglobin. Sensitivity of the assay was 1.71 U/mL, specificity 89%, while coefficient of variation was lower than 2% [39]. 

#### 2.4.3. Assay of Glutathione Peroxidase (GPx) Activity in Erythrocyte

All used reagents: 0,1 M phosphoric buffer, pH 7,0 (KH_2_PO_4_, Na_2_HPO_4_x12 H_2_O), EDTA-Na_2_, sodium azide, glutathione reductase, 10 mM GSH, 2,5 mM NADPH + H^+^, NaHCO_3_, 12 mMtert-butyl hydroxide, 1.5 mM hydrogen peroxide, transforming reagent (4.5 mM KCN; 0.45 mM K_3_Fe(CN)_6_) - were purchased from Sigma-Aldrich. A reactive mixture (phosphoric buffer, glutathione reductase, GSH, NADPH + H^+^, hemolysate with transforming reagent), was incubated for 10 min at 37 °C. Glutathione peroxidase activity was analysed using a UV/VIS Lambda 40 spectrophotometer (Perkin-Elmer). After the incubation, a reaction was initiated by adding tert-butyl hydroxide (or hydrogen peroxide), and a decrease of 340 nm absorption was measured. The amount of enzyme that oxidized 1 µmol of GSH (0.5 µmol NADPH+H) in one min was defined as a unit of the enzyme’s activity. Glutathione peroxidase activity was calculated per 1 g of erythrocyte hemoglobin. Sensitivity of the assay was 6 U/L, specificity 94%, while coefficient of variation was lower than 4% [40].

#### 2.4.4. Assay of Glutathion Transferase (GST) Activity in Erythrocyte

Reagents used to measure the activity of glutathione transferase (0.1 M phosphoric buffer, pH 6.5 (NaH_2_PO_4_, Na_2_HPO_4_ x 7 H_2_O), GSH 20 mM, 1-chloro-2.4-dinitrobenzene (CDNB) 20 mM) were purchased from Sigma-Aldrich Sp. z o.o. (Poznań). Samples of erythrocyte hemolysates were defrosted in room temperature. Glutathion’s transferase activity was analysed using UV/VIS Lambda 40 spectrophotometer (Perkin-Elmer). A reactive mixture was combined (phosphoric buffer, GSH, CDNB, hemolysate), and an increase in λ340 nm absorption was measured. The activity of glutathione’s transferase was determined using molar absorption coefficient of synthesized conjugate (Ɛ = 9600 M^−1^cm^−1^) and calculated per 1 g of erythrocytes’ hemoglobin. Sensitivity of the assay was 1.2 U/L, specificity 97%, while coefficient of variation was lower than 2% [41].

#### 2.4.5. Assay of Glutathione Reductase (GSSG-R) in Erythrocyte

The activity of erythrocyte GSSG-R was determined with the spectrophotometric method of Beutler and Yeh [42]. Reaction mixture for glutathione reductase assay consisted of triethanolamine-EDTA buffer, NADPH+H+ and erythrocytes hemolysate. The reaction was started by adding oxidized glutathione and absorbance change recorded at 340 nm (30 °C, five min). The sensitivity of the assay was 0.14 U/L, specificity 94% and coefficient of variation lower than 4%. 

#### 2.4.6. Assay of Erythrocytes’ and Plasma Levels of 8-Isoprostane and TBARS

ELISA kits were used for measuring erythrocyte and plasma levels of 8-isoprostane (Sunredbio, Shanghai, sensitivity: 0.608 ng/L, assay range: 0.7–180 ng/L), TBARS (Sunredbio, Shanghai, sensitivity: 0.486 nmol/mL, assay range: 0.5–100 nmol/mL) according to the manufacturer’s protocol. Each sample measured in erythrocyte was calculated per 1 g of erythrocyte hemoglobin.

#### 2.4.7. Assay of GSH and GSSG Levels in Erythrocyte 

Erythrocyte levels of GSH (Sunredbio, Shanghai, sensitivity: 6.105 mg/L, assay range: 7–1800 mg/L) and GSSG (Sunredbio, Shanghai, sensitivity: 2.735 mg/L, assay range: 3–600 mg/L) were measured according to the manufacturer’s protocol. Each result was calculated per 1 g of erythrocyte hemoglobin.

### 2.5. Statistical Analysis

Statistical analysis was performed with STATISTICA software (ver. 12.5 PL). In addition to descriptive statistics (median, upper and lower quartiles, minimum and maximum), the normality of distribution of the analysed parameters was determined using the Shapiro-Wilk test. For the values showing the distribution deviating from the normal one, comparison between intervention was done with the Kruskal-Wallis one-way ANOVA analysis of variance and the Mann-Whitney U test was used for comparison between sexes. To carry out the above statistical analyses, the significance level of *p* < 0.05 was adopted. Data are presented as median, upper quartile (Q75) and lower quartile (Q25).

## 3. Results

### 3.1. Body Weight Changes

During the study, significant changes in the body weight of rats exposed to swimming training exercise were observed. Table 1 presents mean rat body weight values before and after the eight-week training program.

The nature of changes in the body weight of rats exposed to swimming training exercise in cold water were described in detail in our previous study [43]. The most interesting observation was that the female rats exposed to spontaneous swimming at 5 °C were the only group in which an increase in body weight occurred during the experimental weeks in relation to baseline values. 

### 3.2. Sex-Specific Differences in the Concentration of Lipid Peroxidation Biomarkers in Erythrocyte

Significance of sex-specific differences and the concentrations of the analysed lipid peroxidation biomarkers in respective groups are shown in Table 2.

The lowest TBARS values, out of all animals, were found in old-sedentary female rats. Erythrocyte TBARS concentrations in the group of male rats swimming at 5 °C were lower compared to the control group and significantly lower (by 41%) in relation to group of the male rats exposed to swimming training exercise at 36 °C. Inversely, significantly higher erythrocyte TBARS concentrations were observed in the female rats of the two training groups compared to the control group (by 83.3% in the cold group and by 72.7% in the thermoneutral group, respectively). 

Significantly higher erythrocyte 8-isoprostane concentrations were found in the two groups of swimming male rats compared to the control groups (by 72.5% and 74.6%, respectively). No significant inter-group differences were found in plasma TBARS and 8-isoprostane levels in the male rats. As opposed to the male rats, the highest values of this parameter and, additionally, high erythrocyte 8-isoprostane concentrations were found in the group of female rats exposed to swimming training exercise in cold water (by 71.9%, compared to the control group). In addition, these female rats were characterised by the highest plasma TBARS concentrations (by 18.1% in relation to the control group).

In the control groups, significantly lower values of erythrocyte TBARS concentration in the female rats (by 79.2%) were shown with no sex-specific differences in plasma levels of this parameter, but with concomitantly higher (by 27.7%) plasmas 8-isoprostane concentrations, in relation to the male rats. Swimming training exercise induced a significantly higher increase in plasma TBARS concentration in the two groups of female rats compared to the male rats, which resulted in the emergence of sex-specific differences, on average by 20.5% of its value in the cold group and by 16.6% in the thermoneutral group. Moreover, erythrocyte TBARS concentrations in the female rats increased significantly following the swimming training exercise in cold water, exceeding the values observed in the male rats by 48.9%. In addition, the significance of sex-specific differences in the case of plasma 8-isoprostane concentrations increased in the two groups exposed to swimming training exercise.

### 3.3. Changes in the Antioxidant Enzymes Activities in Erythrocytes

The level of enzyme activities and sex-specific differences are presented in Table 3. 

Significantly lower glutathione peroxidase (GPx) and glutathione transferase (GST) activities were found in the cold group of male rats compared to the thermoneutral group of male rats (by 95.7% and 41.5%, respectively). The level of GPx activity in this group was also significantly lower compared to the control group (by 98.5%). The two groups of swimming male rats were also characterised by lower glutathione reductase (R-GSSG) activity compared to the control group (by 66.7% in the cold group and by 60% in the thermoneutral group, respectively). However, there were no significant differences in the activity of superoxide dismutase (SOD1) and CAT between all groups of the male rats. In contrast, the only significant difference in the level of enzyme activities between the groups of female rats concerned SOD1. In the group exposed to swimming training exercise in cold water, a significantly higher activity of this enzyme (by 13.4%) was found compared to the control group.

When evaluating the sex-specific differences, significantly higher GPx and R-GSSG activities were observed in the group of female rats swimming at 5 °C compared to the male rats swimming in cold water (by 95% and 68.8%, respectively).

### 3.4. Female Sex Determined GSH Level in Erythrocytes

The GSH and GSSG concentrations and GSH/GSSH ratio in respective study groups, taking into account sex-specific differences, are compared in Table 4.

In this study, no significant differences were found in GSH concentration between the groups of male rates exposed to swimming training exercise at different water temperatures or compared to the control group. On the other hand, a significantly higher concentration of GSH was observed in the female rats of the group swimming in cold water (by 15.4% compared to the control group and by 20.5% in relation to the group of female rats swimming at 36 °C). At the same time, the group of these female rats was characterized by significantly higher GSH concentration, by 30.8%, compared to the male rats training in water of the same temperature.

In the case of GSSG concentration and GSH/GSSG ratio, GSSG concentration was significantly higher only in the control group of sedentary male rats, thus affecting reduction of the value of GSH/GSSG ratio.

## 4. Discussion

Exposure to short-term cold stimuli was used as a form of body hardening to prevent diseases, as well as anti-ageing procedure. However, the effects of cold-water immersion are still not fully understood. It is known to decrease skin, subcutaneous and muscle temperatures and stimulate cutaneous receptors causing vasoconstriction (via sympathetic fibres), which reduces swelling and inflammation by slowing the metabolism and production of metabolites, thereby limiting the degree of injury, and improve neural and muscle recovery [44]. 

### 4.1. The Mechanisms of Adaptive Defence in Response to Thermal Stress

It is known that prolonged exposure to cold increases the rate of metabolism in mammals, which leads to the overgrowth of metabolically active tissues, such as brown (BAT) and white (WAT) adipose tissue, liver, kidneys, small intestine and heart [36,45,46]. The results obtained, relating to sex-specific differences in the nature of adaptive changes, are consistent with the report by Davies et al. [32], who found that female rats maintain body weight better than male rats. Exposure to ambient temperatures below thermal comfort is a thermal stress for the body and, when repeated, can trigger responses of an adaptive nature, which are in line with the assumption of the hormesis phenomenon. Low temperatures combined with exercise provoke increased production of heat accompanied by increased respiration, oxygen consumption and production oxygen species (ROS), which induce cellular oxidative damage, disturb the redox state and can change the activity of antioxidative enzymes. Free radical production and oxidative stress may contribute to the oxidant damage proposed to play a role in the ageing process [11]. ROS, when present at low levels, are involved in intracellular signal transduction. Activation of the receptor of certain interleukins results in increased ROS levels in its vicinity. Moreover, multiple signalling pathways are activated and different cellular responses are induced upon treatment of cells with ROS or ROS-generating xenobiotics. One of these pathways is the synthesis of prostanoids, tissue hormones, derivatives of polyunsaturated fatty acids, synthesised mainly from arachidonic acid [47,48,49]. ROS oxidise proinflammatory lipids and cause inflammation to develop, leading to atherosclerosis [50,51].

It was demonstrated that cold exposure leads to an increase in lipid peroxidation in tissues. For example, in the study by Dede et al. [52], acute hypothermia produced a damage to the cell membrane leading to increased MDA levels. On the other hand, the repeated exposure to low level of oxidative stress may result in some adaptive changes in organisms that help them to resist stress-induced damage. Siems et al. [53] demonstrated the improved antioxidative protection of organism as a result of adaptive response to repeated cold-induced oxidative stress. Repeated exposure to cold is also known to increase aerobic enzyme activities and tissue oxygen consumption in rodent, but its effect on the antioxidant defense system is not fully explained [54,55,56]. Kim et al. suggested that the VEGF gene may be a major regulatory factor in cardiac and skeletal muscle adaptation to the cold environment stimulating angiogenesis and thermogenesis [57]. The study by Venditti et al. indicated tri-iodothyronine (T3) and thyroxine (T4) as mainly responsible for the changes in the liver mitochondrial population capacity to produce H_2_O_2_ associated with cold [58].

### 4.2. Swimming Training Exercise under Thermal Stress Increases Oxidative Stress and Enhances Adaptive Capacity of Female Rats 

We made effort to determine the combined effects of repeated short-term exercise in cold water on oxidative stress markers and main antioxidant enzyme activity in erythrocytes and plasma in senescent male and female rats as a potential strategy for the improvement of antioxidant capacity. Red blood cells, due to high unsaturated fatty acid content (mainly arachidonic acid) in the inner monolayer of their membrane, are predisposed to damage by ROS [59]. Free radical oxidation of the unsaturated fatty acids may also be induced by the presence of transitional metals ions which increase the peroxidation processes both through increased ROS production (Fenton reaction) and by stimulating disintegration of hydroxyperoxides to proper alkyl and peroxyl radicals [60,61]. Fe^2+^ is a stimulator of these changes, as it may be easily released from transferrin and ferritin in blood. When analysing the results of this study, significant intergroup differences can be seen in the level of lipid peroxidation markers in the erythrocytes of the rats exposed to swimming training exercise under different thermal conditions and the sedentary control, which was not so clearly observed with regard to the plasma values. Erythrocytes are constantly confronted with a barrage of reactive oxygen species (ROS) and must be able to detect the mounting oxidant and respond by elevating its intracellular ROS detoxifying pathways, which require both reducing components and energy, especially during the influence of stress factors.

The results of the present study show sex-related differences in the response to one of the strongest environmental stressors, which is cold. Significantly higher erythrocyte TBARS and 8-isoprostane concentrations found in the female rats participating in swimming training exercise at 5 °C compared to both the group swimming at thermal comfort temperature and the control group may indicate that female rats are more prone to stress related to the effect of low temperatures. At the same time, it was observed that among all study groups it is the female rats swimming at 5 °C where a higher erythrocyte GSH concentration and antioxidant enzyme activities were observed compared to other groups. In the case of the male rats participating in swimming training exercise in cold water, the enzyme activities were significantly lower. This may be a reconfirmation of the greater adaptive capacity—also antioxidant one—of female rats in response to thermal stress compared to male rats. It is postulated that mild stress increases GHS levels, mainly through effects on glutamate cysteine ligase (the rate-limiting enzyme for glutathione biosynthesis). This upregulation in glutathione provides protection from more severe stress and may be a critical feature of preconditioning and tolerance [62]. In our study, this phenomenon was not observed in male rats. At the same time, it is worth noting that significantly higher GSSG concentrations were observed in the erythrocytes of old-sedentary male rats accompanied by a high activity—in relation to other groups—of the enzymes involved in maintaining the GSH/GSSG balance, i.e. glutathione reductase, peroxidase and transferase. A decrease in GSH concentration and an increase in GSSG one may be the result of glutathione participation in reactions with oxidants in the situations of increased oxidative and nitrosative stress in the course of ageing. It is well known that glutathione content decreases with ageing [62]. The ratio of reduced glutathione to oxidised glutathione concentrations (GSH/GSSG) is a measure of the cellular redox state. Lower GSH levels occur in many diseases associated with the ageing process, such as cataracts, Alzheimer’s disease, Parkinson’s disease, atherosclerosis, immunodeficiency diseases, cancer and other. The data showed that the reported influences of cold stress on GSH content in several tissues and organs are varied, probably due to very diverse methods of exposure to cold used in different research. In the study by Teramoto et al. [63], GSH concentration decreased under the influence of a two-hour exposure to cold water at 14—1 6°C. The reduction in GSH and increased GSSG content in the liver and blood after acute cold stress in aged animals have been observed [63]. Further, Barja de Quiroga et al. [64] reported that increased GSH levels were present in brown adipose tissue of cold-acclimated rats, and Ohtsuka et al. found that acute cold stress did increase erythrocyte GSH content in humans [65].

In our study, an increased level of erythrocyte GSH concentration with increasing TBARS and 8-isoprostane concentrations in the female rates exposed to low temperatures may indicate, on the one hand, an elevated level of cellular stress, while on the other hand, may be evidence of efficient antioxidant mechanisms of red blood cells. Moreover, the female rats of the group swimming at the same temperature were characterised by higher GPx and R-GSSG activities. As in the experiment of Barja de Quiroga [64], where the level of antioxidant enzyme activity was examined in the rats exposed to cold for 21 days, an increase in GHS concentration was observed, accompanied by increased GPx, SOD and CAT activities, in interscapular brown adipose tissue compared to controls. It was suggested that activation of BAT by repeated cold exposure is accompanied by a large increase in the mitochondrial generation of oxygen radicals, together with augmentation of the preferential substrates of lipid peroxidation and also involves adaptations that compensate for an increased liability to tissue peroxidation. This is accomplished by increases in all of the antioxidant enzymes and in GSH levels. On the contrary, Kaushik and Kaur [28] have shown that the decreased GPx activities in the heart and liver of the cold-stimulated rats indicate the highly reduced capacity to scavenge hydrogen peroxide produced in these tissues in response to cold stress [29].

In the study by Spasić et al. [66], a dependence of the changes in GPx, R-GSSG, GST, CAT and SOD activities on the duration of exposure to low environmental temperature (4 °C) was observed. 

### 4.3. The Reorganization of Antioxidant Defence System under Thermal Stress as a Mechanism of Adaptive Capacity

The changes observed in the analysed components of the antioxidant defense system suggest that a reorganisation occurred in the activity of this system at the molecular level, but these changes are proportional to the duration of exposure. The reduction of GSH occurs due to the reaction catalysed by glutathione reductase (R-GSSG) [67] and is essential for homeostasis in glutathione metabolism due to the fact that many metabolic reactions in organism is associated with oxidation of its molecule, while in erythrocytes this reaction is important not only to maintain the balance between redox processes but also to ensure the availability of nitrogen oxide [68]. In our study, the male rats of the experimental group swimming at 5 °C showed decreased GPx, R-GSSG and GST activities not only compared to the female rats but also to the group swimming at 36 °C and the control old-sedentary male rats (particularly significant for GPx). In addition to a direct antioxidant role for GSH, this thiol is involved as a substrate for GPx and is thus needed for the detoxification of lipid peroxides by the enzyme. Therefore, an observation can be made that the rate and intensity of changes of an adaptive nature depends not only on the duration of exposure but also on sex differences.

SOD is the main protection against the negative effects of superoxide anion which it converts into hydrogen peroxide [69]. The formed H_2_O_2_ is then a substrate for CAT, the expression of which as an inductive enzyme depends on the factors stimulating the formation of H_2_O_2,_ and thus on the activation of SOD. Although CAT is widely distributed in tissues and cells of mammalian organisms, it is the mammalian erythrocytes that are characterised by its very high content [70]. However, the results obtained in this study show no changes in CAT activity following cold training stress. The aforementioned study [65] showed that the activity of this enzyme in cold-exposed animals increased only after 75 and 105 days of cold exposure. It can therefore be assumed that eight-week exposure time is sufficient for CAT activity upregulation.

Estimation of the SOD activity was chosen as an important index to evaluate the contents of oxidative stress injury and antioxidant repair [71]. It was noticed that SOD activity is consistently shown to increase with training in an intensity-dependent manner. Although, as reported in the literature, Mn-SOD (SOD2) is primarily responsible for the observed increase in SOD activity, whereas CuZn-SOD (SOD1) activity is little affected [72], in the present study, a significantly higher erythrocyte activity of SOD1 was found in the group of female rats swimming at 5 °C compared to the control group.

In the study by Siems et al. [53] on the effect of cold on the human body, a higher activity of SOD and CAT, but not GPx, was observed in winter swimmers compared to people who had never participated in winter swimming. In this study, a group of 36 °C winter swimmers was not analysed for possible sex-related differences. An increment in SOD activity suggests that cold stress in female rats may increase the oxidative stress and the rate of O^2−^ formation, and subsequently H_2_O_2_ formation, more than in male ones. Although many studies suggest increased SOD activity after exposure to stress, it is known that antioxidant defense is tissue-specific and may be influenced by the type of stress and its duration [28,73]. Yuksel et al. showed an increased activity of SOD in the lungs and kidneys of rats in response to exposure to cold [74]. On the other hand, Sahin and Gumuslu observed, among others, an increased activity of SOD in the brain of rats [75]. In another experiment, increased SOD levels were observed in BAT of cold-acclimated rats Rattus Norvegicus [67] compared to controls. A significantly increased erythrocyte SOD and an increased GPx activity have been also observed in cold acclimated rats; however, short-term cold acclimation only resulted in a slight rise in erythrocyte SOD activity but a significant decrease in GPx activity [76]. Moreover, previous research shown that in rats exposed to for very low temperature (−60 °C; −90 °C), when compared to controls, the increased in activity of SOD and CAT in the liver and erythrocyte have been observed, as an adaptive response to oxidative stress [77].

## 5. Conclusions

In our study, a significant effect of the physical exercise in low water temperature on changes in the oxidant/antioxidant balance in an organism was observed as a result of repetition of the nondamaging mild stress factors, with a greater effect of exposure compared to the physical exercise of the same intensity but under thermal comfort conditions. At the same time, based on the results obtained, it can be assumed that there are sex-dependent differences in the effects of ageing on lipid peroxidation. Female rats seem to be better adapted to changing thermal conditions of the environment, developing morphological—but also antioxidant—defense mechanisms, mainly in the form of increased erythrocyte SOD activity and GSH concentration, to restore the pro-oxidant/oxidant balance of organism. On the other hand, the organisms of ageing male rats show a reduced capacity to increase the metabolic response to low temperatures compared to female ones.

The findings suggest that systemic exposure to low temperatures is on the one hand a stressor that causes increased pro-oxidant activity of organism, while on the other hand, after taking into account an appropriate duration of exposure, can lead to an adaptive response, particularly efficient in female rats.

## Figures and Tables

**Table 1:** Mean body weights of rats that participated in the study.

Group	Experimental Group (5 °C)		Experimental Group (36 °C)		Old-Sedentary Control Group (C)	
Sex	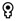	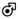	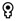	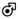	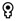	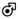
T_0_ [g]	252.5 ± 19.8	530.3 ± 60.2	278.3 ± 33.6	537 ± 75.8	290 ± 26.5	445 ± 57
T_1_ [g]	256.7 ± 16.1	487.1 ± 80.2	262.5 ± 34.9	511 ± 73.4	287 ± 39.9	435 ± 82.6

T_0_ [g]—body mass before experiment; T_1_ [g]—body mass after experiment; 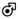
—male sex; 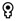
—female sex.

**Table 2:** Erythrocyte and plasma TBARS and 8-isoprostane concentrations in experimental groups (5 °C and 36 °C), control group and sex-specific differences.

Lipid Peroxidation Biomarkers		TBARS RBC [nmol/gHb]		TBARS Plasma [nmol/ml]		8-Isoprostan RBC [ng/gHb]		8-Isoprostan Plasma [ng/L]	
		M	Q25 Q75	M	Q25 Q75	M	Q25 Q75	M	Q25 Q75
Experimental Group (5 °C)	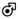	13.8 * 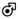 36°	12.7 19.1	23.3	16.7 24.2	37.8 ** 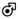 C	33.7 44.5	21.1	20.6 22.3
	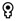	27.0 ** 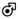 5° ** 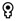 C	22 29.6	29.3 ** 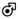 5° * 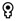 C	28.1 30	44.1 * 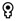 36° ** 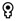 C	21.1 53.4	29.5 ** 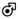 5°	28.6 29.9
Experimental Group (36 °C)	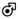	23.4	16.9 26.3	23.6	22.1 25.9	41.0 * 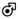 C	40.1 48.8	22.5	21.9 24.1
	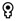	16.5 ** 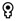 C	13.3 28.7	28.3 ** 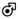 36°	27.1 29	17.2	14.6 52	28.9 ** 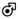 36°	27.6 30.4
Old-sedentary Control Group (C)	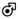	21.6	16.2 27.4	22.3	22.2 22.5	10.4	4.7 16.8	21.6	19.8 23.8
	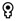	4.5 ** 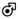 C	4.2 4.8	24	22.3 27.3	12.4	11.1 22.4	29.3 * 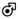 C	29 29.4

Significance of differences between groups at: * *p* < 0.05; ** *p* < 0.01. M—median; Q25—lower quartile; Q75—upper quartile; 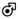
—male sex; 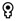
—female sex.

**Table 3:** Erythrocyte superoxide dismutase (SOD1), catalase (CAT), glutathione peroxidase (GPx), glutathione reductase (R-GSSG) and glutathione transferase (GST) activities in experimental groups (5 °C and 36 °C), control group and sex-specific differences.

Antioxidant Enzymes		SOD1 [U/gHb]		CAT [U/g Hb]		GPx [U/g Hb]		R-GSSG [U/g Hb]		GST [U/g Hb]	
		M	Q25 Q75	M	Q25 Q75	M	Q25 Q75	M	Q25 Q75	M	Q25 Q75
Experimental Group (5 °C)	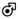	713.6	614.2 791.3	43.5	28.5 58.5	0.01 * 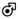 36° * 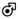 C	0.01 0.08	0.05 * 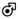 C	0.03 0.08	0.31 * 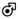 36°	0.2 0.45
	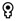	770.8	760.8 810.0	48.6	34.9 57.2	0.2 *** 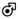 5°	0.13 0.48	0.16 * 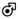 5°	0.09 0.21	0.58	0.32 0.84
Experimental Group (36 °C)	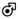	682.0	642.6 697	59.3	37.5 115.8	0.23	0.13 0.38	0.06 * 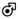 C	0.05 0.10	0.53	0.41 0.72
	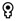	728.0 ** 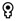 C	645.2 762.1	34.6	23.9 63.2	0.11	0.11 0.36	0.14	0.06 0.16	0.46	0.23 0.52
Old-sedentary Control Group (C)	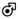	640.9	619.3 719.5	61.2	50.2 82.9	0.67	0.31 1.31	0.15	0.11 0.21	0.72	0.49 1.22
	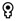	667.9	623.6 694.5	32.6	26.7 32.7	0.13	0.11 0.15	0.12	0.1 0.12	0.52	0.4 0.69

Significance of differences between groups at: * *p* < 0.05, ** *p* < 0.01; *** *p* < 0.001). M—median; Q25—lower quartile; Q75—upper quartile; 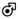
—male sex; 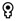
—female sex.

**Table 4:** Erythrocyte GSH and GSSG concentrations and GSH/GSSG ratio in experimental groups (5 °C and 36 °C), control group and sex-specific differences.

Parameters		GSH [μmol/g Hb]		GSSG [μmol/g Hb]		GSH/GSSG ratio	
		M	Q25 Q75	M	Q25 Q75	M	Q25 Q75
Experimental Group (5 °C)	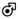	2.7	1.7 3.5	0.9 * 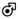 C	0.6 1.4	2.5	1.9 4.0
	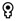	3.9 *** 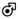 5° * 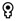 C	3.8 4.2	1.0	0.6 1.6	3.7	2.5 5.9
Experimental Group (36 °C)	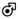	3.2	1.8 3.6	0.6 * 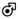 C	0.6 1.3	3.2	2.5 6.1
	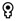	3.1 ** 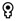 5°	2.0 3.2	0.8	0.5 1.3	2.5	2.5 4.5
Old-sedentary Control Group (C)	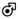	2.4	1.4 3.7	1.3	1.3 1.3	1.9	1.1 2.9
	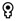	3.3	3.2 3.4	1.0	0.9 1.1	3.5	2.9 3.5

Significance of differences between groups at: * *p* < 0.05, ** *p* < 0.01; *** *p* < 0.001. M—median; Q25—lower quartile; Q75—upper quartile; 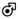
—male sex; 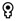
—female sex.
